# Comparison between elementary flux modes analysis and 13C-metabolic fluxes measured in bacterial and plant cells

**DOI:** 10.1186/1752-0509-5-95

**Published:** 2011-06-20

**Authors:** Marie Beurton-Aimar, Bertrand Beauvoit, Antoine Monier, François Vallée, Martine Dieuaide-Noubhani, Sophie Colombié

**Affiliations:** 1 LaBRI, Univ. Bordeaux, UMR 5800. 351, cours de la Libération. F-33405 Talence Cedex, France; 2 INRA Bordeaux, Univ. Bordeaux. UMR 1332 - Fruit Biology and Pathology BP 81. 71 Avenue Edouard Bourlaux. F-33140 Villenave d'Ornon, France

## Abstract

**Background:**

^13^C metabolic flux analysis is one of the pertinent ways to compare two or more physiological states. From a more theoretical standpoint, the structural properties of metabolic networks can be analysed to explore feasible metabolic behaviours and to define the boundaries of steady state flux distributions. Elementary flux mode analysis is one of the most efficient methods for performing this analysis. In this context, recent approaches have tended to compare experimental flux measurements with topological network analysis.

**Results:**

Metabolic networks describing the main pathways of central carbon metabolism were set up for a bacteria species (*Corynebacterium glutamicum*) and a plant species (*Brassica napus*) for which experimental flux maps were available. The structural properties of each network were then studied using the concept of elementary flux modes. To do this, coefficients of flux efficiency were calculated for each reaction within the networks by using selected sets of elementary flux modes. Then the relative differences - reflecting the change of substrate *i.e*. a sugar source for *C*. *glutamicum *and a nitrogen source for *B*. *napus *- of both flux efficiency and flux measured experimentally were compared. For both organisms, there is a clear relationship between these parameters, thus indicating that the network structure described by the elementary flux modes had captured a significant part of the metabolic activity in both biological systems. In *B*. *napus*, the extension of the elementary flux mode analysis to an enlarged metabolic network still resulted in a clear relationship between the change in the coefficients and that of the measured fluxes. Nevertheless, the limitations of the method to fit some particular fluxes are discussed.

**Conclusion:**

This consistency between EFM analysis and experimental flux measurements, validated on two metabolic systems allows us to conclude that elementary flux mode analysis could be a useful tool to complement ^13^C metabolic flux analysis, by allowing the prediction of changes in internal fluxes before carbon labelling experiments.

## Background

Metabolic pathway analysis has become increasingly important to assess inherent network properties in reconstructed biochemical reaction networks [1]. Metabolic Flux Analysis (MFA) provides information on cell responses to environment or genetic perturbations taking into account the regulation of the enzymes and the availability of the substrates. ^13^C Metabolic Flux Analysis (^13^C-MFA), which was developed many years ago, has been used for metabolic engineering especially in microorganisms and has also become an important area of research in the animal or plant research field. The use of a 13C labelled substrate associated with a steady state MFA is probably the most useful and straightforward approach to quantify fluxes in the central metabolism. It is based on the re-distribution of labelling among intermediate metabolites of the network, measured using either NMR for the determination of carbon enrichment and positional isotopomers, or Mass Spectrometry (MS) for the determination of mass isotopomers. Specialized software is available to quantify fluxes in a complex metabolic network [2,3]. ^13^C-MFA has been shown to be an efficient tool to model and quantify the functioning metabolism of cultured prokaryotic and eukaryotic cells and even cultured plant tissues. For instance, in metabolic engineering, ^13^C-MFA has been used to compare the lysine-producing *Corynebacterium glutamicum *grown either on glucose, fructose [4] or sucrose [5]. In plants, this approach has been successfully used to quantify the fluxes of the intermediary metabolism [6] in maize root tips [7,8], tomato cells [9], *Brassica napus *embryos [10-13], sunflower embryos [14] and *Arabidopsis *cells [15].

More theoretically, Elementary Flux Mode (EFM) analysis, a constraint-based approach also called structural analysis, is used to identify all genetically independent pathways that are inherent in a metabolic network. EFM analysis provides a rigorous formalism for describing and assessing metabolic pathways at steady state. It involves three basic conditions, pseudo steady state, feasibility, and non-decomposability [16]. In other words, an EFM is the minimal biochemical pathway that, at steady state, catalyses a set of reactions between input and output metabolites. Its ability to assess the functional and structural properties of metabolic networks means that EFM analysis is suitable for both biotechnology and physiology [17]. EFM analysis has already been used for microbiological systems in metabolic engineering [1]. The first reported application of elementary mode analysis to a biological system in metabolic engineering was in the optimization of the production of 3-deoxy-arabino heptulose phosphate in *E*. *coli *[18]. With improvements in software for EFM analysis, more complicated metabolic networks of different microorganisms have been analyzed *in silico *to design efficient and robust strains to produce desired products. Recently, a classification method for EFMs based on motif finding (named ACoM) has been developed in order to analyze the results of the EFM analysis [19]. In plants, EFM analysis has been successfully applied to the Calvin cycle [20], sucrose metabolism [21] and starch metabolism [22]. However, it is usually stated that EFMs do not take into account the kinetics of the reactions and, therefore, are unable to properly describe the dynamic behaviour of metabolic pathways. The question of the existence of a relationship between EFMs and metabolic flux in real biological systems therefore arises.

In most metabolic studies, objective functions (such as ATP production or cell growth maximization) are used for EFMs to steer the process of metabolic engineering. With the aim of overproducing a target compound, objective functions are based on reaction participation, which links the occurrence of a reaction to the production of the target compound. However, while the criterion of overproduction or efficiency is often significant for optimization (or maximization) studies, this criterion is not entirely meaningful in describing cell physiology without *a priori *objective/selection. For instance, in plant cells and tissues, central carbon metabolism is often dedicated to the production of structural compounds (lipids, proteins...) and storage compounds (starch, sugars, free amino acids, organic acids...).

In our study, we used EFM analysis to compare the structural properties of the metabolic networks with the fluxes experimentally determined by 13C-MFA. To do this, flux maps already published for two biological systems were used: (1) a microbial system, *Corynebacterium glutamicum*, widely used to produce amino acids (especially lysine and glutamate) for which metabolic fluxes have been determined for three carbon sources (glucose, fructose and sucrose) and (2) a plant system, *Brassica napus *embryos, for which metabolic fluxes have been measured in embryos growing in the presence of either mineral (ammonium/nitrate) or organic (alanine and glutamine) nitrogen, in addition to glucose [23]. By calculating coefficients of flux efficiency (derived from a coefficient introduced by [17]) from the EFM matrix, we intended to show a relationship between the measured metabolic fluxes and the coefficient of flux efficiency, in response to changes in the carbon and nitrogen sources for *C*. *glutamicum *and *B*. *napus*, respectively. Then, the robustness of the relationship was tested by enlarging the metabolic network of *B*. *napus *to generic network of heterotrophic plant cells. Finally, the limits of this approach to fit some specific fluxes are discussed.

## Methods

EFM analysis starts with the network representation of the constraints, including network topology, stoichiometries and reaction reversibility [24].

### Two metabolic systems and their maps of fluxes

**Figure 1:**
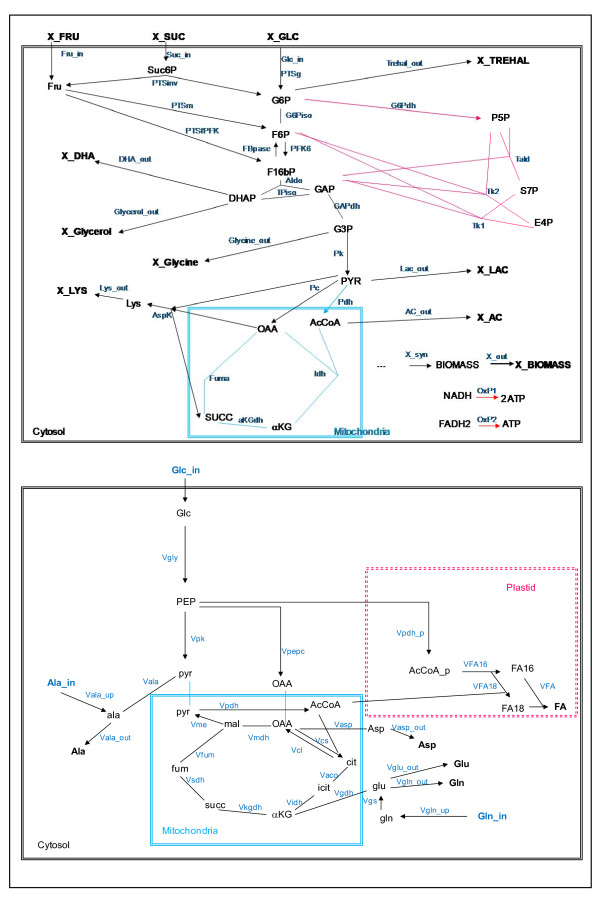
**Metabolic networks of *Corynebacterim glutamicum *(a) and *Brassica napus *(b) corresponding to the experimental fluxes measured by **[4,5]**and **[22]**respectively**. Each colour indicates a pathway: blue for the TCA cycle, black for glycolysis and pink for the PPP. External metabolites are in bold. Irreversible reactions are indicated by unidirectional arrows.

Data obtained from two biological systems were selected from the literature for their relevant map of fluxes determined by 13C-MFA, namely, (1) the microbial system of *Corynebacterium glutamicum *(strain ATCC21526) growing on three different sources of sugars, glucose, fructose [4] or sucrose [5]; (2) The plant system of *Brassica napus *embryos growing on two nitrogen sources, amino acids and ammonium/nitrate [23]. The experimental results obtained in these two biological systems were used to build their metabolic networks by assembling the list of reactions. The metabolic networks are represented in Figure 1 and described as lists of stoichiometric equations (additional file 1). The carbon sources or sinks (nutrients, waste products, stored and excreted products and precursors for further transformation) were defined as external metabolites (input and output). The other compounds were internal metabolites. The reactions were defined as reversible according to their respective equilibrium constant and when exchange fluxes, if available, had been experimentally measured.

In the case of *C.glutamicum*, three distinct networks for each case (glucose, fructose or sucrose) were created to take into account both the differences in the sugar uptake and in the yield of biomass synthesis with respect to the nature of the sugar. These three networks consist of 29, 30 and 31 reactions (thereby 10 reversible reactions) in the presence of glucose, fructose and sucrose respectively. The networks contain 31 metabolites in the presence of glucose and fructose and 32 metabolites in the presence of sucrose (thereby 10 external metabolites) listed in additional file 1.

For *B*. *Napus*, the influence of the substrate on the metabolic network, either with only glucose (ammonium and nitrate as nitrogen source) or with glucose plus amino acids (glutamine and alanine) was analysed. This metabolic network contains 26 reactions (9 reversible) and 30 metabolites (16 internal), mainly located in the TCA pathway (Figure 1b and reactions listed in additional file 1).

### EFM computation

The next step of constraint-based modelling consists in exploiting the network to confine the cellular phenotype with a set of feasible states, i.e. all steady-state flux distributions that lie in a convex space of possible solutions [25]. Methods based on convex analysis make it possible to investigate the convex vector space. The ray of the flux cone corresponds to the minimal functional units of metabolism, also described as EFMs. Their combination characterizes all possible behaviours [26-28]. This may be described as a minimal set of reactions that, at steady state, catalyse some reactions between input and output metabolites with characteristic stoichiometry.

From the tools available for EFM computation, CellNetAnalyzer http://www.mpi-magdeburg.mpg.de/projects/cna/cna.html a free package providing a user-friendly environment for structural and functional analysis of biochemical networks based on MATLAB (Mathworks, Inc.) and the new implementation of METATOOL algorithm (*elmo *http://elmocomp.sourceforge.net/elmocomp.shtml) were chosen for computation [29].

The computation step performed on *C*. *glutamicum *networks provides three EFMsets, named *Sg*, *Sf *and *Ss *with only glucose, fructose and sucrose respectively as carbon sugar substrate in the medium and containing 212, 365 and 884 EFMs respectively. Note that the size of the *Ss *EFMset is higher than the size of *Sg *and *Sf *as the sucrose is metabolized in both glucose and fructose.

The computation step performed on *B*. *napus *network led to 51 modes. As this set of EFMs included EFMs involving both glucose and amino acids, two subsets of EFMs were built, selected from only glucose (ammonium and nitrate as nitrogen source) and from both glucose plus amino acids (glutamine and alanine) as a carbon substrate. The first EFMset, named *Nm*, contained 15 EFMs that use only glucose as a carbon substrate in the medium (*Glc_up*, medium with an inorganic source of nitrogen). The second EFMset, named *Naa*, contained 50 EFMs in which at least one of the uptake reactions is present (glucose *Glc_up*, glutamine *gln_up *or alanine *ala_up*).

Interestingly, although the number of reactions in slightly lower in the *B*. *napus *network than in the *C*. *glutamicum *networks, the number of EFMs is much lower in the *B*. *napus *network than in the *C*. *glutamicum *networks. This shows the decrease in the level of the complexity of the *B*. *napus *network mainly involving the TCA pathway. For both biological systems, each EFMset represents a functional network with specific behaviour in which the coefficients of flux efficiency of the reactions are calculated.

### The coefficient of flux efficiency

To address the question of whether the function and/or regulation of a complex metabolic network can be predicted by elementary mode analysis, our analysis relies on the calculation of a coefficient called "flux efficiency". The coefficient of flux efficiency is determined from the parameter of 'control-effective fluxes' introduced by [17] characterize both flexibility (ability of cellular systems to adapt to a wide range of environmental conditions) and the efficiency of the metabolic fluxes in the network related to the growth or the production of ATP. But, in the metabolism of plant cells or plant tissues for instance, it is not relevant to formulate an objective such as the maximization of growth or ATP production. Here, therefore, the coefficient of flux efficiency is determined from the matrix of EFMs without taking into account a specific target. As defined by [17], the flux efficiency of each mode is related to the investment required to produce the enzyme needed to establish the mode, approximated by the sum of all absolute fluxes, because for comparable metabolite concentrations, the flux through a reaction scales linearly with the enzyme concentration. The coefficient of flux efficiency is calculated from the matrix of EFMs of *N *elementary modes (rows) and *P *reactions (columns). First, the EFM matrix containing the elements *e_i,j _*(row *i *and column *j*) is transformed to an efficiency matrix *Mε *(same size as the matrix of EFMs) by assigning a value of efficiency  to each flux in the mode:(1)

Then, the flux efficiency  of a reaction *j *is determined as the average of the flux efficiencies through this reaction in the EFM matrix (size N):(2)

Note that the forward and the backward reactions (with positive and negative values respectively in the EFM matrix) are considered separately in order to assess the efficiency of each reaction specifically. The efficiency of a reversible reaction is thus the sum of its positive value (forward reaction) and its negative value (backward reaction).

The coefficient of flux efficiency depends above all both on the occurrence of the reactions in the EFMset and on the size of the EFMs. That means that the coefficient of flux efficiency is equal to zero when a reaction does not happen and, conversely, a highly frequent reaction in relatively short EFMs is affected by a high value of flux efficiency. The coefficient of flux efficiency of each reaction of the five EFMsets (*Sg, Sf *and *Ss *for *C*. *glutamicum *and *Nm *and *Naa *for the *B*. *napus*) was calculated. With respect to a reference condition, i.e. glucose condition for *C*. *glutamicum*, and organic nitrogen condition for *B*. *napus*, the relative difference (*Δ*) of both parameters, i.e. the measured flux and the flux efficiency, was calculated. The relative difference, quantifying the perturbation due to the change of substrate, was expressed as the ratio between the difference generated by the change in the carbon (*C*. *glutamicum*) or nitrogen (*B*. *napus*) source, at the numerator, and the absolute value of the reference, at the denominator. The relative difference of measured fluxes (*ΔF*) was:(3)

with *F_ref _*and *F_c _*the metabolic fluxes measured in the reference condition (glucose for *C*. *glutamicum*, and organic nitrogen for *B*. *napus*) and in the other state, respectively. Similarly, the relative difference of flux efficiencies  was calculated between  and  the flux efficiencies in the reference condition (glucose for *C*. *glutamicum*, and organic nitrogen for *B*. *napus*) and in the other state, respectively.

## Results

In this section, we compare the EFM analysis with the experimental results obtained for two systems, *Corynebacterium glutamicum *cultured with three carbon sources (glucose, fructose or sucrose) [4,5] and *Brassica napus *embryos grown on two nitrogen sources (ammonium/nitrate or amino acids) [14,23].

### Main experimental results

The comparison of the central metabolism of lysine-producing *Corynebacterium glutamicum *(strain ATCC 21526) grown on glucose and fructose [4] shows: (1) a significant decrease in the yield of lysine and biomass production on fructose compared to glucose, (2) marked differences in intracellular flux namely a decreased flux through the Pentose Phosphate Pathway (PPP) (14.4% instead of 62.0% of total substrate uptake) and an increased flux through the pyruvate dehydrogenase (PDH) and the TCA cycle (95.2% instead of 62% of the total substrate uptake) on fructose as compared to glucose. On sucrose [5], the flux through the Pentose Phosphate Pathway (PPP) is relatively high (55.7%) as the glucose monomer of sucrose is channelled into the PPP and flux through the TCA is high (78.2% normalized to an uptake of hexose units of 100%). The fructose residue was mainly taken up by the fructose-specific phosphotransferase system (PTS) and entered glycolysis at the fructose-1,6-bisphosphate level. Glucose-6-phosphate isomerase operates in the gluconeogenetic direction from fructose-6-phosphate to glucose-6-phosphate, and supplies additional carbon from the fructosyl of sucrose to the PPP [5].

In non-photosynthetic tissues such as *B*. *napus *embryos, while sugar is the main carbon source, amino acids in phloem cells (usually glutamine and alanine) provide an additional carbon source for growing tissues. In the *Brassica napus *embryos, the main difference between the organic (amino acids, i.e. alanine and glutamine) and the inorganic (ammonium/nitrate) nitrogen condition was the redirection of fluxes leading into and out of the TCA cycle [23]. When the embryos were provided with amino acids, the pyruvate kinase flux was reduced, probably because of a decrease in the demand for carbon to build amino acids. Glutamine is incorporated in the TCA cycle as a second anaplerotic pathway. Consequently, PEPC activity, which catalyses the main anaplerotic pathway when sugar is the only carbon source, is reduced whereas the malic enzyme is activated. Cyclic flux through the TCA cycle (i.e. through isocitrate dehydrogenase and aconitase) is null in the presence of organic nitrogen.

### Comparison between EFM analysis and experimental results

**Figure 2:**
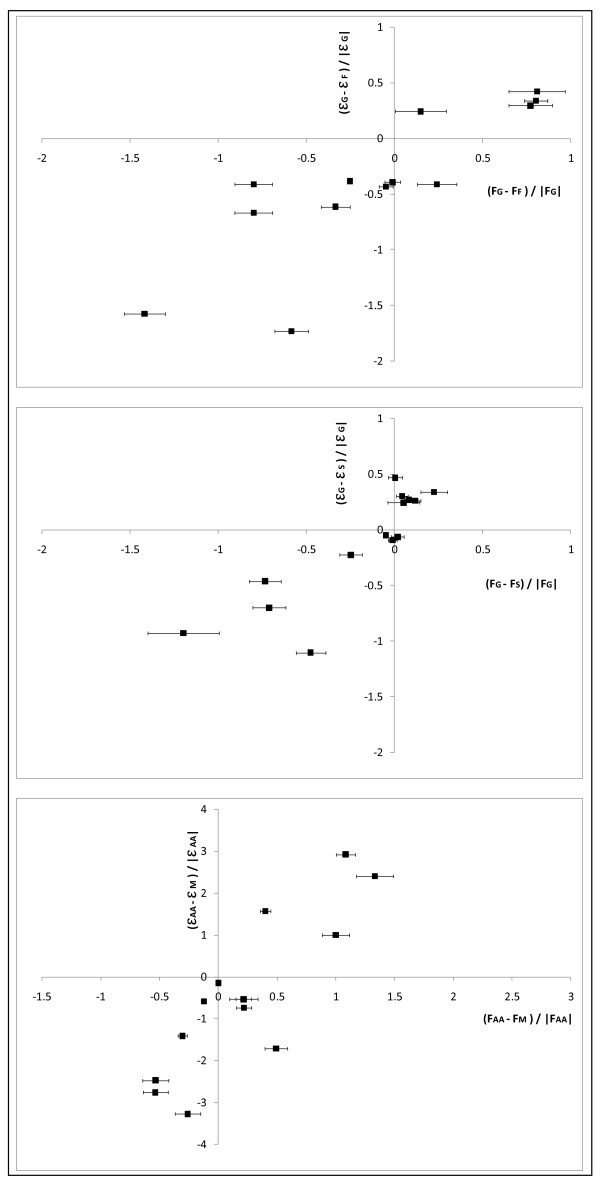
**Relative differences of the measured fluxes (*ΔF*) and of the flux efficiencies  for bacteria (a, b) and plant cells (c)**. The coefficients of flux efficiency  were calculated according to the Materials and Methods from the matrix of EFMs computed from the networks described in figures 1a (*C*. *glutamicum *) and 1b (*B*. *napus)*. The fluxes measured on *C*. *glutamicum *grown in the presence of different carbon sources (glucose, fructose, sucrose) and on *B*. *Napus *embryos grown in the presence of mineral (ammonium plus nitrate) or organic (amino acids) nitrogen sources, were compiled from [4,5] and [22] respectively. For each reaction, the relative difference of the flux efficiency  and that of the measured flux (*ΔF*) was expressed taking the glucose (*Sg*, *C*. *glutamicum*, a, b) and the organic nitrogen (*Naa*, *B*. *napus*, c) conditions as references.

EFM analysis was performed on both biological systems, a bacterial system (*C*. *glutamicum *[4,5]) and a plant system (*B*. *napus *embryos [14,23]) and the coefficient of flux efficiency was calculated for each reaction of the networks, as described in the Material and Methods section. The results of the EFM analysis were compared with the experimental results obtained by taking the glucose condition as a reference condition for both organisms. Then, the influence of a change in the carbon (*C*. *glutamicum*) and the nitrogen source (*B*. *napus*) was analysed by comparing  with *ΔF *through the respective reaction. These relative  and *ΔF *(with standard errors calculated from experimental data) were plotted in Figure 2 and the values listed in additional file 2. The Wilcoxon non-parametric test (p-value = 0.323) confirms that we can accept the assumption that there is no difference between these two sets of parameters (**and *ΔF*) for both the *C*. *Glutamicum *and *B*. *napus *systems. This result means that at a high *ΔF *(or a low *ΔF*) corresponds a high  (or a low ) in the considered EFMset.

### *C. glutamicum*

**Figure 3:**
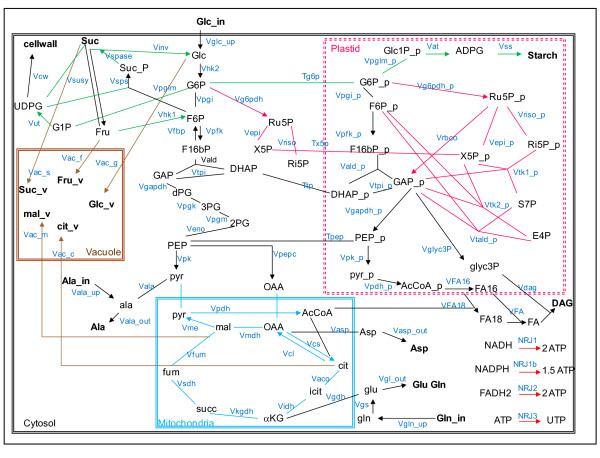
**The enlarged metabolic network of a heterotrophic plant cells**. Each colour indicates a pathway: blue for the TCA cycle, black for glycolysis and also for the fluxes towards output metabolites, pink for the PPP, green for the sucrose and starch synthesis, red for respiration and brown for storage in vacuole. External metabolites are in bold. Irreversible reactions are indicated by unidirectional arrows.

For *C*. *glutamicum *(Figure 3a and 3b), the fluxes towards the output metabolites are not taken into account (see the discussion below). Concerning the uptake fluxes, the relative difference cannot be calculated as the three sugar uptakes involve specific reactions (see additional file 2). Concerning the internal fluxes (14 on fructose and 15 on sucrose) most of them have a  (from the EFM analysis) similar to that of *ΔF*. Indeed, as regards 14 of the 15 internal fluxes determined in the network (black squares in Figure 2a and 2b), in agreement with experimental results, the EFM analysis predicts correctly: (1) a negative  of phospho-glucose isomerase (*PGI*), fumarase (*fum*) isocitrate dehydrogenase (*Idh*), and alpha-keto dehydrogenase (*aKGdh*), pyruvate dehydrogenase (*Pdh*) and (2) a positive of  aspartokinase (*aspK*) and of all enzymes of the PPP (*G6Pdh, tald, tk1 and tk2*). It is notable that on the one hand *PGI *exhibits the lowest change in both the measured fluxes and the flux efficiencies, while on the other hand, the PPP fluxes (*G6Pdh, tald, tk1 and tk2) *exhibit the highest change, regardless of the carbon source (fructose and sucrose). Nevertheless, some discrepancies occur for some enzymes (Figure 3a and 3b and additional file 2). For instance, even if the sign of the relative difference is similar for isocitrate dehydrogenase (*Idh*), the value of  is far from *ΔF*, both under fructose and sucrose conditions (compared to the glucose condition). Moreover, on fructose (compared to glucose) pyruvate carboxylase (*PC*) displays a negative  while the *ΔF *is positive. Finally, on sucrose (compared to glucose) the  of the phosphofructokinase (*PFK*) is positive while the *ΔF *was close to zero. In summary, EFM analysis correctly predicts both the sign and the amplitude of the change in fluxes when the sugar source is changed in *C*. *glutamicum*.

### *B. napus*

In the *B*. *napus *embryos (Figure 2c), the flux of the isocitrate dehydrogenase (*Vidh*) was not included in our analysis because its flux measurements yield large error bars (more than 300%) (see additional file 2). Concerning the fluxes towards the output metabolites (named with the suffix '_out'), they are assumed to vary little since the experimental values are standardized according to the same growth rate (biomass flux). Accordingly, EFM analysis shows only slight differences in  of these fluxes towards the output metabolites. Concerning the uptake fluxes (named with the suffix '_up'), the sign of both  and *ΔF *is similar: positive for alanine and glutamine uptake (*ala_up *and *gln_up *superimposed) and negative for glucose uptake (*Glc_up*). Concerning the nine internal fluxes determined around the TCA cycle (Figure 2c and details in additional file 2), in agreement with experimental results, the EFM analysis displays a negative  for pyruvate kinase (*Vpk*), phosphoenolpyruvate carboxylase (*Vpepc*), ATP citrate lyase (*Vcl*) and citrate synthase (*Vcs*) and a positive  for glutamate dehydrogenase (*Vgdh*), transaminase (*Vala*), fumarase (*Vfum*) and glutamate synthase (*Vgs*). Note that the fluxes involved in nitrogen assimilation, i.e. glutamate dehydrogenase (*Vgdh*) and glutamate synthase (*Vgs*), exhibit the highest changes in both measured fluxes and flux efficiencies. Among the 16 fluxes measured experimentally (9 internal, 3 uptake fluxes and 4 fluxes towards output metabolites), the direction of the change (sign of *ΔF *) of 15 fluxes are accurately predicted by the change in flux efficiencies  of the EFM analysis, and only one, the malic enzyme flux (*Vme*) is not. To resume, EFM analysis correctly predicts both the sign and the amplitude of the change in fluxes when the nitrogen source is changed for *B*. *napus*.

The robustness of the previous observations was further tested by adding reactions in the core metabolic network of *B*. *napus*, giving a large metabolic network describing the central metabolism of heterotrophic plant cells.

### Comparison between EFM analysis on an enlarged network and results of *B. napus A network of heterotrophic plant cells*

The three metabolic networks of *C*. *glutamicum *involve the main pathways of the central carbon metabolism. In these networks, cofactors such as ADP/ATP, NADP/NADPH and NAD/NADH (defined as internal metabolites) are balanced, thus constraining the EFMs not only through the carbon balance but also through the redox and energetic status. In contrast, the fluxes of *B*. *napus *embryos were measured on a core network centralized around the TCA cycle without balancing ADP/ATP and NAD/NADH. While the EFM analysis fits the experimental results on this core network, we wonder what would happen to the correlation if the whole metabolic network of heterotrophic plant cells was taken into consideration.

The complete metabolic network of the *B*. *napus *embryos (see Figure 3 and details listed in additional file 1) encompasses, not only the TCA cycle (blue), but also the glycolysis (black), the pentose phosphate pathway (pink), the starch and sucrose pathways (green) and the storage in the vacuole (brown) [7,10,12]. Irreversible reactions are indicated by unidirectional arrows. The glycolytic pathway is duplicated so that reversible glycolysis is present in the cytosol and irreversible glycolysis in the plastids, because amyloplasts lack fructose-1,6-bisphosphatase [30,31]. The TCA cycle provides precursors for several biosynthetic processes, such as nitrogen fixation and biosynthesis of amino acids [32]. The pentose phosphate pathway includes the irreversible oxidative branch, whereas the non-oxidative branch is reversible. The enzymes of the oxidative branch, which also exist in the cytosol, can lead to the synthesis of pentose phosphates in both the cytosol and the plastids [33,34]. Sucrose is metabolized in cytosol, whereas starch is metabolized in plastids from imported hexose-phosphates (G1P or G6P). Several effluxes are illustrated for (1) protein synthesis from several amino acids (glutamate, glutamine, aspartate and alanine), (2) lipid synthesis (diacyl glycerol) from plastidial pyruvate and trioses, (3) synthesis of cell wall polysaccharides from UDP-glucose and (4) storage of organic acids (malate, citrate) and sugars (glucose, fructose, sucrose) into the vacuole. The energy reactions (*NRJ*, red arrows in Figure 3) are essential to balance the cofactors of the system. Subcellular compartments, such as mitochondria and plastids, can lead to potentially reversible transport of several metabolites (G6P, pentose-phosphates, PEP and DHAP [35-38]). At steady state, there are 70 different metabolites in this network including 15 external metabolites (exogenous glucose, glutamine, alanine, CO_2_, sugars and organic acids stored in the vacuole, amino acids for protein synthesis, cell wall polysaccharides, starch and lipids). Based on carbon and energy balances, they are assumed not to be limiting in this system. The other small molecules (e.g. oxygen, ammonium, phosphate, pyrophosphate and water) are not included in the metabolic network. The other 55 metabolites, including cofactors (NAD/ NADH, NADP/NADPH, and FAD/FADH2) and ADP/ATP are internal, meaning that they are expected to be balanced at steady state [17]. The direction (reversibility or irreversibility) of the 78 reactions is derived from thermodynamic properties, and 33 reactions are reversible. In cytoplasmic and plastidial glycolysis, the bidirectionality of the reactions and the existence of isoenzymes can increase the number of EFMs. For instance, the conversion of fructose-1,6 biphosphate (F16bP) to fructose-6 phosphate (F6P) is catalyzed only by fructose bisphosphatase (*Vfbp*) and the reversible conversion (from F6P to F16bP) is catalyzed by two enzymes, 6-phosphofructokinase (*Vpfk*) and fructose-6-phosphate phosphotransferase. However, in this network, only one reaction (*Vpfk*) is recorded in order to consider only once the isoenzyme in the EFM computation.

### EFM analysis on this enlarged network and comparison with experimental results of *B. napus*

**Figure 4:**
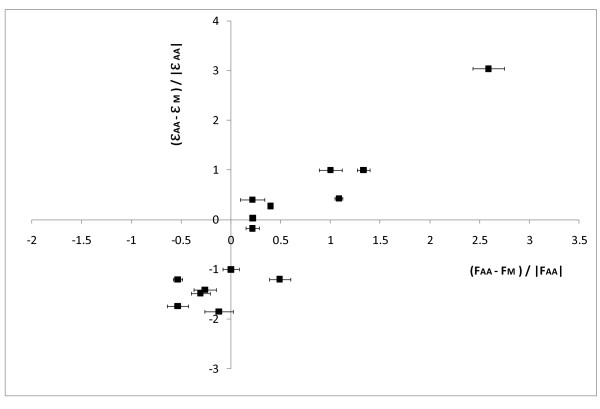
**Relative differences of the measured fluxes (*ΔF*) and of the flux efficiencies  in the enlarged metabolic network of a heterotrophic plant cell**. The coefficients of flux efficiency  were calculated from the matrix of EFMs computed from the enlarged network described in figure 3. For each flux (reaction) measured on *B*. *Napus *embryos grown in the presence of mineral or organic nitrogen sources [22], the relative difference of the flux efficiency  and that of the measured flux (*ΔF*) was calculated taking the organic nitrogen (*LNaa*, *B*. *napus*) condition as reference.

With the enlarged network described above, the result of the computation is a set of 114 614 EFMs, indicating a large number of potentially active metabolic routes at steady state. Two subsets of EFMs were built: the first, named *LNm*, contains 13 481 EFMs that use only glucose as a carbon substrate and the second set, named *LNaa*, contains 113 700 EFMs in which at least one of the uptake reactions is present (glucose *Glc_up*, glutamine *gln_up *or alanine *ala_up*). For these two sets the coefficients of flux efficiency were calculated as previously described in the material and method section. The relative difference of measured fluxes (*ΔF*) was compared with that of the flux efficiency  of the corresponding reaction in the enlarged network (Figure 4 and additional file 2). A statistical analysis (Wilcoxon non-parametric test) confirms that there is no difference between *ΔF *and , thus emphasizing the existence of a relationship between the change in flux efficiencies and the change in measured fluxes. The isocitrate dehydrogenase (*Vidh*) is omitted in the plot for the same reason as above (large errors in the measured fluxes). The overall changes in experimental fluxes observed with respect to the nitrogen source are confirmed by the change in the flux efficiency of the corresponding reaction in the enlarged network. The same conclusions as for the core network of *B*. *napus *can be drawn: (1) almost similar change in the fluxes towards output metabolites; for the uptake fluxes, the change in both, measured fluxes and flux efficiencies are similar; (2) for the internal fluxes, in agreement with experimental results, the EFM analysis displays a negative  of the pyruvate kinase (*Vpk*), the phosphoenolpyurate carboxylase (*Vpepc*), the ATP citrate lyase (*Vcl*) and the citrate synthase (*Vcs*) and a positive  of the glutamate dehydrogenase (*Vgdh*), the transaminase (*Vala*), the fumarase (*Vfum*) and the glutamate synthase (*Vgs*). Note that the fluxes involved in nitrogen assimilation, i.e. glutamate dehydrogenase (*Vgdh*) and glutamate synthase (*Vgs*), have the highest differences in both  and *ΔF*. Again, only the change in malic enzyme flux (*Vme*) is not correctly predicted by the change in flux efficiencies. But in summary, the relationship between the EFM analysis and experimental measurements in *B*. *napus *is confirmed using an enlarged metabolic network of heterotrophic plant cells.

## Discussion

In the present report, an EFM analysis was performed to compare the structural properties of the metabolic networks (mainly in the central carbon metabolism) with the fluxes experimentally determined by ^13^C-MFA using maps of both a microbial system (*C*. *glutamicum*) [4,5] and a plant system (*B*. *napus *embryos) [23]. By calculating the coefficient of flux efficiency from the EFM matrix, we show a clear relationship between this coefficient and the measured metabolic fluxes in response to changes in the carbon and the nitrogen sources for *C*. *glutamicum *and *B*. *napus*, respectively. More precisely, for *C*. *glutamicum*, the change in the internal fluxes measured experimentally (14 on fructose and 15 on sucrose) are accurately predicted by the change in flux efficiencies calculated from the EFM analysis performed for the three sugar sources. For *B*. *napus*, the main changes in experimental fluxes observed using the two nitrogen sources (mineral and amino acids) are confirmed by the change in the flux efficiencies of the corresponding reaction in both the core network and the enlarged network describing the metabolism of a heterotrophic plant cell.

These results establishes a (strong) link between the structure of the network, based on thermodynamic and stoichiometric data, and its metabolic activity, measured by ^13^C labelling experiments, whatever the biological system, microorganism or plant cell. Thus, to some extent, changes in metabolic activity following environmental changes could be predicted without considering kinetics. This result is in agreement with [17] showing that EFM analysis, or more exactly the number of EFMs involved in the biomass synthesis in bacteria, somehow relates structure to function, i.e. topology to activity. Indeed the number of EFMs under a given metabolic condition can be used as a quantitative measure of the degree of freedom, i.e. the flexibility of metabolism. Here, for instance, from the global EFMset calculated on the enlarged network of *B*. *napus*, the number of EFMs utilizing a given carbon substrate shows interesting properties: glucose (13481 EFMs) is used about 3 to 4 times more often than glutamine (3822 EFMs). Moreover, alanine (576 EFMs) is much less used than glucose (13481 EFMs) or glutamine (3822 EFMs). These observations also match biological intuition [17].

It is worth noting that the relationship described above is maintained -and even improved- by enlarging the metabolic network of *B*. *napus*. This confirms and strengthens the link between the metabolic activity measured by ^13^C-labelling experiments and the structure of the network, especially if the network is large and strongly connected. Indeed, the presence of branch points in a network creates multiple routes between different intermediates and this presents one of the largest challenges for EFMs to correlate with 13C-MFA. Nevertheless, several limitations of this approach have to be mentioned. While most of the differences of flux efficiency are well correlated with the experimental fluxes, some of them do not fit the experimental results. For instance, in *C*. *glutamicum*, the flux efficiency of the phosphofructokinase (*PFK*) decreased on sucrose compared to glucose while the experimental flux did not change. Also for the core and the enlarged network of *B*. *napus*, the  of the malic enzyme (*Vme*) is negative while the *ΔF *is positive. Finally, while four fluxes towards output metabolites (fatty acids, aspartate, alanine and glutamate) contribute to the relationship in the *B*. *napus *network, the eight fluxes towards the output metabolites in the *C*. *glutamicum *network are not represented in Figure 2. Whereas the sign of  and *ΔF *is similar for lysine, trehalose, lactate and DHA, the amplitude of the change is rarely similar for these fluxes. In the same way, there is no more coherence between *ΔF *and  for the fluxes towards biomass, glycerol, acetate and glycine on fructose and sucrose compared to glucose (data in additional file 2). If the model behaviour is at variance with expectation, there are two possible causes: either a mistake has been made in the definition of the model, or the expectation is incorrect.

Assuming that the experimental fluxes are properly determined (especially the fluxes towards output metabolites, more easily measured than the internal fluxes), this suggests that a part of the network is not accurately designed. Several hypotheses can be made: (1) the lack of knowledge about both the activity and the compartmentalization of the enzymes impedes the correct representation of the network (2) the fluxes towards macromolecules, such as proteins, nucleic acids and starch, are simplified or inaccurately represented, (3) the small compounds (phosphate, oxygen, nitrogen etc...) which are assumed not to be limiting in the system plays key roles in the network's functionality, (4) the rate of some reactions (enzymes) can be controlled by substrate-dependent kinetics and, in addition, regulated by effectors. The EFM analysis, based on the stoichiometry and the thermodynamic reversibility of reactions, is a useful tool in the structural modelling of a metabolic network at steady state [39]. However, this modelling does not take into account the kinetic properties of the enzymes inherent in the functioning and the regulation of the whole system. A further challenge is thus the reintegration of kinetics data.

Most of the EFM approaches have been carried out on a single model, taking into account the intrinsic correlations between fluxes and their optimization with respect to ATP and biomass synthesis [17,20]. Here, we demonstrate a relationship between the EFMs and the measured fluxes by ^13^C-MFA by comparing the networks generated under different environmental conditions (i.e. changes in carbon and nitrogen sources). The observed relationship is defined foremost by the internal fluxes - highly connected between each other - but does not include the fluxes towards output metabolites.

The relationship between the change in measured fluxes and the change in flux efficiency can be (partially) destroyed by removing one reaction from the network. For instance, in the *C*. *glutamicum *network, the omission of *GAPdh*, *PFK*, and *aldolase *definitely lead to destruction of the network and the omission of *PK *and *AspK *lead to a marked decrease in both the size of the EFMset and the number of fluxes making it impossible to evaluate the relationship previously described (data not shown). In the *B*. *napus *network, only the omission of glucose uptake leads to a total destruction of the network, as expected since the glucose is the reference condition. For all the other fluxes of the networks, their omission only leads to a lower number of EFMs but the relationship between the change in measured fluxes and the change in flux efficiency is maintained. The remaining relationship when one reaction is removed from the network can be explained by the flexibility (plasticity) of the network. This highlights the extreme degree of redundancy in the system and the difficulty in pin-pointing any one enzyme as a 'key' or 'regulatory' step in the absence of a global analysis of the whole system. The use of the model provides a rational basis for identifying those enzymes.

With the whole metabolic network of heterotrophic plant cells, a robust network of high redundancy is obtained. Achieving the ability to work with large models is primarily a technical challenge. A bigger network is synonymous with a better one when the knowledge about the functioning of the cell is available to accurately design the network. It is also difficult to build a generic network without taking into account the specificity of the cell and the tissue (storage of fatty acids for the embryos of *B*. *napus*). However, this work illustrates that EFM analysis -thanks to the coefficient of flux efficiency- could be used as a tool to analyse a large and connected network and, subsequently, to predict changes in metabolic behaviour before undertaking difficult and time-consuming experiments. Similarly, [40] recently showed that is possible to predict the absolute fluxes (estimated by 13C-MFA) and the direction and magnitude of the changes caused by stress conditions using constraint-based modelling based on flux minimization in central carbon metabolism of *Arabidopsis thaliana*. Indeed, while the development of new experimental techniques, such as sequencing and chip technologies, triggered the boom in omics data, fluxomics does not measure more than a dozen fluxes simultaneously.

The present paper illustrates that a significant proportion of the activity of highly connected pathways is controlled by the topology of the network *per se*. This work re-integrates topology analysis as a complementary approach to experimental ^13^C-MFA. As far as we know, this is the first time that ^13^C-MFA experimental results have been compared with EFM analysis performed without objective such as maximization of cell growth or metabolite production. The originality of this work was to develop a network analysis integrating microbial and plant systems, without considering optimization of the metabolism for overproducing a target compound as it is often done in metabolic engineering (see review [41] for state of the art tools for modelling metabolism typically used in the domain of metabolic engineering). Indeed, the majority of the constraint-based metabolic modelling performed until now has focused on unicellular organisms. The generic network from unicellular to multi-cellular organisms, together with constraint-based modelling, represents a key foundational advance in systems biology that is essential for seeking comprehension of biological functioning throughout the integration of data with mathematical models.

## Conclusions

The aim of this study was to investigate the relationship between structural modelling of metabolic network, using EFMs, and experimental data obtained by ^13^C metabolic flux analysis. By using metabolic networks of different origins, i.e. bacterial and plant cells, and sets of flux measured under different environmental conditions, i.e. change in the carbon or the nitrogen source, we have shown that at least the sign, and most often the amplitude, of the changes in internal fluxes are similar to the respective changes in the coefficients of flux efficiency, calculated from the matrix of elementary flux modes. This structural analysis shows specific behaviour in agreement with experimental conclusions; future work will attempt to generalize the potential interest of EFMs and the intrinsic value of the coefficient of flux efficiency to correlate with metabolic fluxes or "omics" data.

## Competing interests

The authors declare that they have no competing interests.

## List of abbreviations

Ac: Acetate; AccoA: acetyl coenzymeA; ADPG: ADP-glucose; aKG: 2-oxoglutarate; Ala: alanine; Asp: aspartate; CellWall: cell wall polysaccharides; Cit: citrate; DAG: Diacyl glycerol; DHA: Dihydroxyacetone; DHAP: Dihydroxyacetone phosphate; dPG: diphospho glycerate; 3PG: 3-phospho glycerate; 2PG: 2-phospho glycerate; E4P: erythrose 4-phosphate; FA: fatty acids; FA16 and FA18: fatty acids with a carbon chain of 16 and 18 carbon respectively; FADH2: flavin adenine dinucleotide; F6P: fructose-6-phosphate; Fru: fructose; F16bP: fructose-1,6-biphosphate; Fum: fumarate; GAP: glyceraldehyde-3-phosphate; Glc: glucose; Gln: glutamine; Glu: glutamate; Glyc: glycerol; Glyc3P: glycerol 3-phosphate; G1P: glucose-1-phosphate; G6P: glucose-6-phosphate; Icit: isocitrate; Lac: Lactate; Lys: Lysine; Mal: malate; NADH: nicotinamide adenine dinucleotide; NADPH: nicotinamide adenine dinucleotide phosphate; NMR: nuclear magnetic resonance; OAA: oxaloacetate; PEP: phosphoenolpyruvate; Pyr: pyruvate; P5P: Pentoses-5-phosphate; Ri5P: ribose-5-phosphate; Ru5P: ribulose-5-phosphate; suc: sucrose; succ: succinate; sucP: sucrose phosphate; S7P: sedoheptulose-7-phosphate; Trehal: Trehalose; UDP_Glc: Uridine diphosphate-glucose; UTP: Uridine triphosphate; X5P: xylulose-5-phosphate; '_in': for exogenous metabolites uptake by the cell (glucose, glutamine and alanine); '_out': for exogenous amino acids (glutamine, glutamate, aspartate, alanine); '_p': for metabolites located in plastid; '_v': for metabolites located in vacuole.

## Authors' contributions

MBA and SC designed the study and the manuscript. BB, and MDN participated in the design of the study and helped draft the manuscript, FV performed calculations of EFMs and AM helped draft the manuscript. All authors read and approved the final manuscript.

## Supplementary Material

Additional file 1 **The networks described in *Metatool *file format for EFM analysis**. Click here for file

Additional file 2**Experimental fluxes and the corresponding coefficients of flux efficiency  calculated for both systems *C*. *glutamicum *and *B*. *napus***.Click here for file
