# Supplementary material for: Comparison between elementary flux modes analysis and 13C-metabolic fluxes measured in bacterial and plant cells
Source: BMC Syst Biol. 2011 Jun 20;5:95. doi: 10.1186/1752-0509-5-95 (PMC3148577; doi:10.1186/1752-0509-5-95)
Supplement: Additional file 1 — The networks described in Metatool file format for EFM analysis. [file 1752-0509-5-95-S1.PDF]

Metatool input files

```
#METATOOL input file C. glutamicum /glucose
#*******************

-ENZREV
G6Piso Aldo TPiso GAPdh Idh aKGdh Fuma Tald Tk1 Tk2

-ENZIRREV
Glc_in PTSg PFK6 Pk Pc Pdh G6Pdh OxP1 OxP2 AspK Trehal_out Ac_out Lac_out
Glycerol_out Glycine_out DHA_out X_syn X_out Lys_out

-METINT
AccoA aKG ADP ATP BIOMAS DHAP E4P FAD FADH2 F6P F16bP Glc G6P GAP 3PG LYS
NAD NADH OAA P5P Pyr S7P SUCC NADP NADPH

-METEXT
Glc_in BIOMAS_out LYS_out TREHAL_out AC_out LAC_out Glyc_out Glycine_out
DHA_outCO2

-CAT
Glc_in : Glc_in = Glc .
PTSg : Glc = G6P .
PFK6 : F6P + ATP = F16bP + ADP .
G6Piso : F6P = G6P .
Aldo : F16bP = GAP + DHAP .
TPiso : DHAP = GAP .
GAPdh : GAP + NAD + ADP = NADH + 3PG + ATP .
Pk : 3PG + ADP = ATP + Pyr .
Pc : Pyr + CO2 + ATP = OAA + ADP .
Pdh : Pyr + NAD = AccoA + CO2 + NADH .
Idh : AccoA + OAA + NADP = aKG + NADPH + CO2 .
aKGdh : aKG + NAD + ADP = SUCC + CO2 + NADH + ATP .
Fuma : SUCC + NAD + FAD = FADH2 + OAA + NADH .
G6Pdh : G6P + NADP + 2 NADP = P5P +  CO2 + 2 NADPH   .
Tald : 2 P5P = S7P + GAP .
Tk1 : S7P + GAP = F6P + E4P .
Tk2 : P5P + E4P = F6P + GAP .
OxP1 : 2 NADH + 4 ADP = 4 ATP + 2 NAD .
OxP2 : 2 FADH2 + 2 ADP = 2 ATP + 2 FAD .
AspK : OAA + Pyr + 2 NADPH  + ATP = LYS + CO2 + SUCC + 2 NADP + ADP .
Trehal_out : 2 G6P + ATP = TREHAL_out + ADP .
Glycerol_out : DHAP + NADH = Glyc_out  .
Glycine_out : 3PG = Glycine_out + NADH .
DHA_out : DHAP = DHA_out .
Lys_out : LYS = LYS_out  .
Ac_out : AccoA + ADP =  AC_out  + ATP .
Lac_out : Pyr + NADH = LAC_out + NAD .
X_syn : 11 G6P + 4 F6P + 48 P5P + 15 E4P + 7 GAP + 60 3PG + 108 Pyr + 93
aKG + 49 OAA + 135 AccoA + 19 LYS + 50 NADPH  + 1500 ATP = 500 BIOMAS  + 70
CO2 + 50 NADP + 1500 ADP.
X_out : BIOMAS = BIOMAS_out .
```

```
#METATOOL input file C. glutamicum fructose.dat
#*******************

-ENZREV
G6Piso Aldo TPiso GAPdh Idh aKGdh Fuma Tald Tk1 Tk2

-ENZIRREV
Fru_in PTSm PTSfPFK FBPase Pk Pc Pdh G6Pdh OxP1 OxP2 AspK Trehal_out Ac_out
Lac_out Glycerol_out Glycine_out DHA_out X_syn X_out Lys_out

-METINT
AccoA aKG ADP ATP BIOMAS DHAP E4P FAD FADH2 FRU F6P F16bP G6P GAP 3PG LYS
NAD NADH OAA P5P Pyr S7P SUCC NADP NADPH

-METEXT
X_FRU BIOMAS_outLYS_out TREHAL_out AC_out LAC_out Glyc_out Glycine_out
DHA_out CO2

-CAT
Fru_in : X_FRU = FRU .
PTSm : FRU = F6P .
PTSfPFK : FRU + ATP = F16bP + ADP .
G6Piso : F6P = G6P .
FBPase : F16bP = F6P .
Aldo : F16bP = GAP + DHAP .
TPiso : DHAP = GAP .
GAPdh : GAP + NAD + ADP = NADH + 3PG + ATP .
Pk : 3PG + ADP = ATP + Pyr .
Pc : Pyr + CO2 + ATP = OAA + ADP .
Pdh : Pyr  + NAD = AccoA + CO2 + NADH .
Idh : AccoA + OAA + NADP = aKG + NADPH  + CO2 .
aKGdh : aKG  + NAD + ADP = SUCC + CO2 + NADH + ATP .
Fuma : SUCC + NAD + FAD = FADH2 + OAA + NADH .
G6Pdh : G6P + 2 NADP = P5P +  CO2 + 2 NADPH  .
Tald : 2 P5P = S7P + GAP .
Tk1 : S7P + GAP = F6P + E4P .
Tk2 : P5P + E4P = F6P + GAP .
OxP1 : 2 NADH + 4 ADP = 4 ATP + 2 NAD .
OxP2 : 2 FADH2 + 2 ADP = 2 ATP + 2 FAD .
AspK : OAA + Pyr + 2 NADPH  + ATP = LYS + CO2 + SUCC + 2 NADP + ADP.
Trehal_out : 2 G6P + ATP = TREHAL_out + ADP .
Glycerol_out : DHAP + NADH = Glyc_out + NAD .
Glycine_out : 3PG  + NAD = Glycine_out + NADH .
DHA_out : DHAP = DHA_out .
Lys_out : LYS = LYS_out  .
Ac_out : AccoA + ADP =  AC_out + ATP .
Lac_out : Pyr + NADH = LAC_out + NAD .
X_syn : 6 G6P + 2 F6P + 25 P5P + 8 E4P + 4 GAP + 37 3PG + 57 Pyr + 49 aKG +
46 OAA + 71 AccoA + 10 LYS + 50 NADPH  + 1500 ATP = 500 BIOMAS  + 70 CO2 +
51 NADP + 1500 ADP .
X_out : BIOMAS = BIOMAS_out.
```

```
#METATOOL input file C. glutamicum sucrose.dat
#*******************

-ENZREV
G6Piso Aldo TPiso GAPdh Idh aKGdh Fuma Tald Tk1 Tk2

-ENZIRREV
Suc_in PTSinv PTSm PTSfPFK PFK6 Pk Pc Pdh G6Pdh OxP1 OxP2 AspK Trehal_out
Ac_out Lac_out Glycerol_out Glycine_out DHA_out X_syn X_out Lys_out

-METINT
AccoA aKG ADP ATP BIOMAS DHAP E4P FAD FADH2 FRU F6P F16bP G6P GAP 3PG LYS
NAD NADH OAA P5P Pyr S7P SUCC SUC6P NADP NADPH

-METEXT
X_SUCROSE BIOMAS_outLYS_out TREHAL_out AC_out LAC_out Glyc_out Glycine_out
DHA_outCO2

-CAT
Suc_in : X_SUCROSE = SUC6P .
PTSinv : SUC6P = G6P + FRU .
PTSm : FRU = F6P .
PTSfPFK : FRU + ATP = F16bP + ADP .
PFK6 : F6P + ATP = F16bP + ADP .
G6Piso : F6P = G6P .
Aldo : F16bP = GAP + DHAP .
TPiso : DHAP = GAP .
GAPdh : GAP + NAD + ADP = NADH + 3PG + ATP .
Pk : 3PG + ADP = ATP + Pyr .
Pc : Pyr + CO2 + ATP = OAA + ADP .
Pdh : Pyr + NAD = AccoA + CO2 + NADH .
Idh : AccoA + OAA + NADP = aKG + NADPH  + CO2 .
aKGdh : aKG + NAD + ADP = SUCC + CO2 + NADH + ATP .
Fuma : SUCC + NAD + FAD = FADH2 + OAA + NADH .
G6Pdh : G6P + 2 NADP = P5P +  CO2 + 2 NADPH  .
Tald : 2 P5P = S7P + GAP .
Tk1 : S7P + GAP = F6P + E4P .
Tk2 : P5P + E4P = F6P + GAP .
OxP1 : 2 NADH + 4 ADP = 4 ATP + 2 NAD .
OxP2 : 2 FADH2 + 2 ADP = 2 ATP + 2 FAD .
AspK : OAA + Pyr + 2 NADPH  + ATP = LYS + CO2 + SUCC + 2 NADP + ADP.
Trehal_out : 2 G6P + ATP = TREHAL_out + ADP .
Glycerol_out : DHAP + NADH = Glyc_out + NAD .
Glycine_out : 3PG + NAD = Glycine_out + NADH .
DHA_out : DHAP = DHA_out .
Lys_out : LYS = LYS_out  .
Ac_out : AccoA + ADP =  AC_out  + ATP .
Lac_out : Pyr + NADH = LAC_out + NAD .
X_syn : 6 G6P + 9 F6P + 24 P5P + 8 E4P + 4 GAP + 35 3PG + 58 Pyr + 33 aKG +
23 OAA + 74 AccoA + 10 LYS + 50 NADPH  + 1500 ATP = 500 BIOMAS  + 70 CO2 +
50 NADP + 1500 ADP .
X_out : BIOMAS = BIOMAS_out.
```

```
#METATOOL input file B. Napus .dat
#*******************

-ENZREV
Vaco Vidh Vkgdh Vsdh Vfum Vmdh Vala Vgdh Vgs

-ENZIRREV
Vgly ala_up Vgln_in Vpk Vcl Vpepc Vpdh Vcs Vme VFA16 VFA18 VFA Vpdh_p
Vgln_out Vglu_out Vasp_out Vala_out

-METINT
PEP OAA AccoA cit icit aKG succ fum mal pyr AccoA_p FA16 FA18 ala glu gln

-METEXT
Glc_in gln_in ala_in ala_out asp_out glu_out gln_out CO2 FA ADP ATP NAD
NADH NADP NADPH FAD FADH2

-CAT
ala_up : ala_in = ala .
Vgly : Glc_in + 2 NAD = 2 PEP + 2 NADH .
Vpk : PEP + ADP = ATP + pyr .
Vpepc : CO2 + PEP = OAA .
Vpdh : pyr + NAD = CO2 + AccoA + NADH .
Vcl : cit + ATP = OAA + AccoA + ADP .
Vcs : OAA + AccoA = cit .
Vaco : cit = icit .
Vidh : icit + NAD = aKG + CO2 + NADH .
Vkgdh : aKG + NAD = CO2 + NADH + succ .
Vsdh : succ + FAD = FADH2 + fum .
Vfum : fum = mal .
Vmdh : mal + NAD = NADH + OAA .
Vme : mal + NAD = CO2 + NADH + pyr .
Vala : pyr + glu = ala + aKG .
Vgdh : aKG + NADH = glu + NAD .
Vgln_in : gln_in = gln .
Vgs : gln + aKG + NADPH = 2 glu + NADP .
Vglu_out : glu = glu_out .
Vgln_out : gln = gln_out .
Vasp_out : OAA + glu = asp_out + aKG .
Vala_out : ala = ala_out .
Vpdh_p : PEP + NAD + ADP = ATP + CO2 + AccoA_p + NADH .
VFA16 : 8 AccoA_p + 7 ATP + 14 NADPH = FA16 + 14 NADP + 7 ADP .
VFA18 : AccoA + FA16 + ATP + NADPH = FA18 + NADP + ADP .
VFA : FA16 + 2 FA18 = 3 FA .
```

```
#METATOOL input file
#*******************
# The enlarged network of heterotrophic plant cells HPC.dat


-ENZREV
Vpgi Vald Vtpi Vgapdh Vpgk Vpgm Veno Vaco Vidh Vkgdh Vsdh Vfum Vmdh Vepi
Vriso Vald_p Vtpi_p Vpgi_p Vepi_p Vriso_p Vtk1_p Vtk2_p Vtald_p Tg6p Ttp
Tpep Tx5p Vpglm Vpglm_p Vsusy Vala Vasp Vgdh

-ENZIRREV
Glc_up ala_up gln_up Vhk1 Vhk2 Vpfk Vfbp Vpk Vcl Vpepc Vpdh Vcs Vg6pdh
Vg6pdh_p Vpfk_p Vgapdh_p Vpk_p Vrbco Vpdh_p Vme Vat Vss Vut Vinv Vsps
Vspase Vgs Vcw VFA16 VFA18 VFA Vdag Vglyc3P NRJ1 NRJ1b NRJ2 NRJ3 Vgl_out
Vasp_out Vala_out Vac_g Vac_f Vac_s Vac_m Vac_c

-METINT
Suc Glc G6P F6P F16bP DHAP GAP dPG 3PG 2PG PEP OAA AccoA cit icit aKG succ
fum mal pyr Ru5P X5P Ri5P F6P_p G6P_p F16bP_p GAP_p DHAP_p Ru5P_p X5P_p
Ri5P_p S7P_p E4P_p PEP_p pyr_p AccoA_p UDPG ADPG G1P G1P_p SucP Fru ADP ATP
UTP NAD NADH NADP NADPH FAD FADH2 FA16 FA18 FA glyc3P ala asp glu gln

-METEXT
Glc_in gln_in ala_in CellWall DAG starch ala_out asp_out gl_out CO2 Glc_v
Fru_v Suc_v mal_v cit_v

-CAT
Glc_up : Glc_in = Glc .
ala_up : ala_in = ala .
gln_up : gln_in = gln .
Vpgi : G6P = F6P .
Vhk1 : ATP + Fru = F6P + ADP .
Vhk2 : ATP + Glc = G6P + ADP .
Vpfk : ATP + F6P = F16bP + ADP .
Vfbp : F16bP = F6P .
Vald : F16bP = DHAP + GAP .
Vtpi : DHAP = GAP .
Vgapdh : GAP + NAD = dPG + NADH .
Vpgk : dPG + ADP = 3PG + ATP .
Vpgm : 3PG = 2PG .
Veno : 2PG = PEP .
Vpk : PEP + ADP = ATP + pyr .
Vpepc : CO2 + PEP = OAA .
Vpdh : pyr + NAD = CO2 + AccoA + NADH .
Vcl : cit + ATP = OAA + AccoA + ADP .
Vcs : OAA + AccoA = cit .
Vaco : cit = icit .
Vidh : icit + NAD = aKG + CO2 + NADH .
Vkgdh : aKG + NAD = CO2 + NADH + succ .
Vsdh : succ + FAD = FADH2 + fum .
Vfum : fum = mal .
Vmdh : mal + NAD = NADH + OAA .
Vme : mal + NAD = CO2 + NADH + pyr .
Vg6pdh : G6P + NADP = CO2 + Ru5P + NADPH .
Vepi : Ru5P = X5P .
Vriso : Ru5P = Ri5P .
Vpgi_p : G6P_p = F6P_p .
Vg6pdh_p : G6P_p + NADP = CO2 + Ru5P_p + NADPH .
Vepi_p : Ru5P_p = X5P_p .
```

```
Vriso_p : Ru5P_p = Ri5P_p .
Vtk1_p : S7P_p + DHAP_p = Ri5P_p + X5P_p .
Vtk2_p : F6P_p + DHAP_p = E4P_p + X5P_p .
Vtald_p : S7P_p + DHAP_p = E4P_p + F6P_p .
Vpfk_p : ATP + F6P_p = F16bP_p + ADP .
Vald_p : F16bP_p = GAP_p + DHAP_p .
Vtpi_p : GAP_p = DHAP_p .
Vgapdh_p : DHAP_p + NAD + ADP = ATP + NADH + PEP_p .
Vpk_p : PEP_p + ADP = ATP + pyr_p .
Vrbco : Ru5P_p + CO2 = 2 DHAP_p .
Vpdh_p : pyr_p + NAD = CO2 + AccoA_p + NADH .
Tg6p : G6P = G6P_p .
Ttp : DHAP = DHAP_p .
Tpep : PEP = PEP_p .
Tx5p : X5P = X5P_p .
Vpglm_p : G1P_p = G6P_p .
Vat : G1P_p + ATP = ADPG + ADP .
Vss : ADPG = starch .
Vpglm : G1P = G6P .
Vut : G1P + UTP = UDPG .
Vsusy : Fru + UDPG = Suc .
Vinv : Suc = Fru + Glc .
Vsps : F6P + UDPG = SucP .
Vspase : SucP = Suc .
Vala : pyr + glu = ala + aKG .
Vasp : OAA + glu = asp + aKG .
Vgdh : aKG + NADH = glu + NAD .
Vgs : gln + aKG + NADPH = 2 glu + NADP .
Vgl_out : glu = gl_out .
Vasp_out : asp = asp_out .
Vala_out : ala = ala_out .
Vcw : UDPG = CellWall .
VFA16 : 8 AccoA_p + 7 ATP + 14 NADPH = FA16 + 14 NADP + 7 ADP .
VFA18 : AccoA + FA16 + ATP + NADPH = FA18 + NADP + ADP .
VFA : FA16 + 2 FA18 = 3 FA .
Vglyc3P : DHAP_p + NADH + ATP = glyc3P + NAD + ADP .
Vdag : glyc3P + ATP + 2 FA = DAG + ADP .
Vac_g : Glc = Glc_v .
Vac_f : Fru = Fru_v .
Vac_s : Suc = Suc_v .
Vac_m : mal = mal_v .
Vac_c : cit = cit_v .
NRJ1 :  NADH + 2 ADP = 2 ATP .
NRJ1b : 2 NADPH + 3 ADP = 3 ATP + 2 NADP .
NRJ2 : FADH2 + 2 ADP = 2 ATP + FAD .
NRJ3 : ATP = UTP + ADP.
```
